# Using thermokinetic methods to enhance properties of epoxy resins with amino acids as biobased curing agents by achieving full crosslinking

**DOI:** 10.1038/s41598-024-54484-0

**Published:** 2024-02-22

**Authors:** Melissa Walter, Marcel Neubacher, Bodo Fiedler

**Affiliations:** Institute of Polymers and Composites, Hamburg University of Technology, Hamburg, Germany

**Keywords:** Mechanical properties, Composites, Sustainability

## Abstract

Fibre-reinforced polymers (FRPs) are used in numerous industrial sectors and contribute to reducing CO_2_ emissions due to their outstanding properties in lightweight design. However, sustainable alternatives must be developed since the matrix polymers utilised contain substances hazardous to health and the environment. In widely used epoxy resins, the curing agents are mainly critical. Using biomolecules instead of synthetic curing agents can significantly reduce composites' toxicity and petrol-based carbon content. This study considerably exceeds the thermo-mechanical properties of epoxies cured with amino acids described in the literature until now. It demonstrates competitive or even better properties than state-of-the-art epoxies cured with petrol-based amine curing agents. For instance, the tensile strength of arginine-cured epoxy is more than twice as high as reported before and 13.5% higher compared to the petrol-based reference. At the same time, a high elongation at break of over 6% was accomplished, making these polymers suitable as matrix materials in FRPs. Furthermore, the glass transition onset of up to 130 °C is sufficiently high for many applications. The key to success is the development of individual curing profiles based on thermokinetic analysis. The work provides the development and analysis of several biomolecule-cured epoxies with promising property spectra.

## Introduction

Current economic and social challenges like climate change, as well as dependencies and resulting scarcity of resources, lead to a variety of scientific tasks. In the field of plastics technology, sustainable, environment- and health-friendly alternatives for petrochemical systems are in demand. In the field of FRPs, the properties of bio-based alternatives are currently still mostly below the requirements for their typical use in mobility applications e.g. aircraft structures and in rotor blades of wind turbines for energy generation^1^. The FRPs used for these applications consist of continuous fibres, usually made of glass or carbon. Recently, natural fibres, such as flax fibres, are also increasingly used^2^. The fibres in the form of scrims or fabrics have the function of absorbing the occurring forces. These are embedded in a polymer matrix which transfers the loads to the fibres. Epoxy resins are often used to fulfil this purpose, because of their outstanding properties^3^. They consist of at least one resin component with epoxy groups and a minimum of one curing agent. The curing agents are often particularly critical for the environment and health, which is why it is reasonable to find alternatives for this component^3^. Because of their chemical structure, amino acids and their derivatives offer potential as biobased curing agents. These are obtained from the fermentation of proteins^4^, available at low cost, permitted as food supplements and are therefore harmless to health.

It has already been shown in the literature that amino acids are capable of curing epoxy resins^5^,^6^. So far, only configurations with accelerators have been published. However, the DGEBA resins cured with L-arginine and 2-Ethyl-4-methyl-imidazole in^6^ and with UR500 (1,1'-(4-Methyl-1,3-phenylene)bis(3,3-dimethylurea)) in^7^ were brittle and relatively weak. The modulus reached so far (> 3 GPa) is adequate for use in load-bearing composite applications, but the tensile strengths of 43 MPa / 56 MPa at elongations of break of 1.67% / 2.65% appear insufficient. The reason is, that the required strain to failure of the matrix polymer in FRPs should be higher than the fibre rupture strain (e.g. glass fibres ≈ 5%^8^). In addition, the strength of the matrix must be sufficiently high to allow the load introduction from the matrix into the fibres on a micro-mechanical level. Characteristic properties depend strongly on the degree of curing and the used temperature profile^9,10^. Therefore, this study aims to determine optimised and tailored temperature profiles for curing novel FRPs by using thermokinetic methods to make amino acid-cured epoxies ready to apply on an industrial level.

Curing can be achieved with many different temperature profiles composed of the parameters temperature, time and heating as well as cooling rates. In each case, the conversion rate differs. To determine a suitable temperature profile, analytical methods of varying complexity exist that describe the reaction kinetics by solving the kinetic Eq. (1) ^11^, where α is the degree of conversion, *t* the time, *T* the temperature, *k*(*T*) the reaction rate constant and *f*(*α*) the conversion function. Equation (1) includes the Arrhenius equation^12^ to determine *k*(*T*). A is the pre-exponential factor, *E*_*A*_ the activation energy and *R* the universal gas constant.

1 $$\frac{d\alpha }{{dt}} = {\Phi }\left( {T,\alpha } \right) = k\left( T \right) \cdot f\left( \alpha \right) = A \cdot exp\left( {\frac{{ - E_{A} }}{RT}} \right) \cdot f\left( \alpha \right)$$

Different methods can be used to determine the parameters *A*, *f*(*α*) and $E_{A}$. Fundamentally, a distinction is made between isoconversional and isokinetic methods. The first mentioned requires the knowledge of the temperatures at which an equivalent reaction stage occurs for different heating rates while the second depends on the reaction model^13,14^. In this study, isoconversional methods are used, because they are considered comparatively superior for the accurate determination of kinetic parameters^14^. They can again be differentiated into differential (e.g. Friedman (FM)) and integral methods (e.g. Kissinger–Akahira–Sunose (KAS) and Ozawa-Flynn-Wall (OFW)). Necessary data can be generated by different experimental methods such as Differential Scanning Calorimetry (DSC), Dielectric Analysis (DEA), Thermogravimetric Analysis (TGA) or rheological measurements.

In the FM method^15^, the main kinetic equation is logarithmised (Eq. (2)) and the so-called FM plot is applied. For this purpose, measurement data are transformed into a coordinate system with ln(dα/dt) over 1,000/T. Points with the same degree of conversion are then connected linear and evaluated as linear function (Eq. 3).

2 $$ln\left( {\frac{d\alpha }{{dt}}} \right) = \ln \left( {A \cdot f\left( \alpha \right)} \right) - \frac{{E_{A} }}{RT}$$

3 $$y = b + a \cdot x$$

Considering Eq. (2), the parameters can be determined with $y = ln\left( {d\alpha /dt} \right)$; $x = - 1/T$; ${\text{a}} = E_{A} /R$ and ${\text{b}} = \ln \left( {A \cdot f\left( \alpha \right)} \right)$ using multiple linear regression analysis.

For the KAS method^16^, the kinetic Eq. (1) is converted into Eq. (4), where β is the linear heating rate. This equation is then integrated (Eq. 5) and logarithmised, resulting in Eq. (6) ^16^.

4 $$\frac{d\alpha }{{dT}} = \frac{1}{\beta } \cdot {\Phi }\left( {T,\alpha } \right) = \frac{1}{\beta } \cdot k\left( T \right) \cdot f\left( \alpha \right) = \frac{A}{\beta } \cdot exp\left( {\frac{{ - E_{A} }}{RT}} \right) \cdot f\left( \alpha \right)$$

5 $$\beta \cdot \mathop \smallint \limits_{0}^{a} \frac{d\alpha }{{f\left( \alpha \right)}} = A \cdot \mathop \smallint \limits_{{T_{0} }}^{T} exp\left( {\frac{{ - E_{A} }}{RT}} \right)dT$$

6 $$ln\left( {\frac{\beta }{{T^{2} }}} \right) = ln\left( {\frac{A}{F\left( \alpha \right)}} \right) - \frac{{E_{A} }}{RT} - ln\left( {\frac{{E_{A} }}{R}} \right)$$

Measurement data of different heating rates are transferred into a so-called KAS plot, into a coordinate system with ln(β/T^2^) over 1,000/T. As with the FM method, points with the same α are connected and the relevant parameters are evaluated via Eq. (3) with $y = ln\left( {\beta /T^{2} } \right)$; $x = - 1/T$; ${\text{a}} = E_{A} /R$ and ${\text{b}} = \ln \left( {A/F\left( \alpha \right)} \right) - {\text{ln}}\left( {E_{A} /R} \right)$ (see Eq. 6).

The Doyle approximation^17^ is used within the OFW method^18^, resulting in Eq. (7).

7 $$ln\left( \beta \right) = 5.3305 + ln\left( {\frac{A}{F\left( \alpha \right)}} \right) - 1.052 \cdot \frac{{E_{A} }}{RT}$$

Using the OFW plot with the ordinate *ln*(*β*) over 1,000/T, the required parameters can also be determined via isoconversional linear functions with $y = ln\left( \beta \right)$; $x = - 1/T$; ${\text{a}} = 1.052 \cdot E_{A} /R$ and ${\text{b}} = 5.3305 \cdot \ln \left( {A/F\left( \alpha \right)} \right)$ (see Eq. 7).

The described methods can represent multiple-step reactions without parallel reaction steps although there is no detailed knowledge about the reactions^19,20^. If parallel and independent reactions take place, only the mean values of $E_{A}$ are considered. The FM method can be applied to isothermal and dynamic measurements, while the KAS and OFW methods require dynamic measurement data^21^. Despite the simplifying assumptions, the presented isoconventional methods can represent epoxy reactions well in many cases^13,14,20^. Especially the FM method is often particularly suitable^22–24^. Over time, further methods were developed, for example, the Kamal-Sourour method^25^ considers non-catalytic and autocatalytic reactions with two constants, but assumes that primary and secondary amines have the same reactivity and no etherification reaction occurs^26^. In addition to the mentioned methods, there are also simpler approaches, e.g., Kissinger (ASTM E2890)^27–29^ or Ozawa (ASTM E698)^30,31^, which determine only one value of $E_{A}$ and A without dependency on α. However, these cannot adequately represent epoxy reactions, which are often autocatalytic^32^.

Consequently, the thermokinetic methods FM, KAS and OFW are the most suitable for generating curing profiles for epoxy resins with amino acids. In this study, amino acids are used without accelerators for the first time. In addition, amino acids that have not yet been utilised as curing agents are also investigated. Remarkably, the resulting material properties fulfil the typical requirements for matrix polymers in load-bearing composite applications. This is attributed to the adjusted constant and slow conversion rate which avoids excessive exothermic heat development^33^, thus preventing thermal degradation^34^. Moreover, complete curing is verified using FTIR and DSC techniques, which additionally provide new insights into the progress of the reactions.

## Materials and methods

### Materials and mixture of the components

In the present study, the petroleum-based resin 827 (*Epikote™ 827*, Westlake, Houston, USA), as well as the partially bio-based resin SR810 (*SR Infugreen 810*, Sicomin, Chateauneuf les Martigues, France, 38 wt% bio-C), were investigated with regard to the curability by several amino acids, namely L-Arg (*L-Arginine*, Buxtrade GmbH, Buxtehude, Germany), L-Phe (*L-Phenylalanine*, Harrison Sport Nutrition S.L., Peligros, Spain) and L-Trp (*L-Tryptophan*, Buxtrade GmbH, Buxtehude, Germany). UR500 (Dyhard® UR500, Alzchem Group AG, Trostberg, Germany) was partly used as an accelerator. The amine curing agent 137H (*EPIKURE™ MGS™ RIMH 137*, Westlake, Houston, USA) was compared for reference.

To determine the respective mixing ratio, the amine hydrogen equivalent weights (AHEW) of the amino acids are theoretically calculated. Therefore, the molecular weight is divided by the amount of active hydrogen atoms in the molecular structure. The stoichiometric ratio was calculated under consideration of the respective epoxy equivalent weight (EEW). The EEW of 827 was taken from the data sheet while the EEW of SR810 was titrated according to standard DIN EN ISO 3001. The mixing ratios of the analysed configurations as well as the bio-C-Fraction are summarised in Table 1. The accelerator component UR500 is added at 1 pph.

**Table 1:** Mixing ratios and biobased fractions of all manufactured configurations.

	Resin (wt.%)	Curing agent (wt.-%)	Accelerator (wt.-%)	Biobased fraction (wt.%)
827/137H	77.78	22.22	0	0
827/L-Arg	87.92	12.08	0	12.08
827/L-Arg/UR500	87.05	11.96	0.99	11.96
827/L-Phe/UR500	76.03	22.98	0.99	22.98
827/L-Trp/UR500	77.34	21.67	0.99	21.67
SR810/137H	76.25	23.75	0	28.98
SR810/L-Arg	86.98	13.02	0	46.07

For an appropriate dispersion of the solid curing agents, the particular curing agents were mortared and dispersed with a three-roll mill 120E (EXAKT Advanced Technologies GmbH, Norderstedt, Germany). The process parameters were determined according to^35^ (see Supplementary Table S1 online). For comparison, the dispersion was prepared 2 × 3 min with a planetary centrifugal mixer ARE-250 (THINKY U.S.A., INC., Laguna Hills, USA) and 2 × 10 min with an ultrasonic bath SONOREX RK 510S (BANDELIN electronic GmbH & Co. KG, Berlin, Germany). Furthermore, the ultrasonic homogeniser SONOPULS 2200.2 (BANDELIN electronic GmbH & Co. KG, Berlin, Germany) with the sonotrode KE 76 in the rosette RZ 3 was compared (10 × 1.5 min intervals, ice bath). The particle dimensions and particle-resin dispersions were analysed using a digital microscope VHX-6000 (Keyence Corporation, Osaka, Japan) with transmitted light.

### Shelf life of the dispersions

The uncured resin is tested rheological with an ARES rheometer (TA Instruments Inc., New Castle, USA) in plate-plate mode according to DIN EN ISO 3219 (20–50 °C, 2 K/min, plate diameter: 40 mm, gap: 0.5 mm, frequency: 5 Hz, shear strain: 10%). Measurements were repeated after 1, 4, 8, 12, 16, 20 and 24 weeks with cooled storage (11,5 ± 0,6 °C) and storage at room temperature. The viscosity at 35 °C is evaluated. For selected systems, namely 827/L-Arg and 827/L-Arg/UR500, DSC measurements after 34 weeks, of room temperature and cooled storage were conducted on a DSC 204 F1 Phoenix (Erich NETZSCH GmbH & Co. Holding KG, Selb, Germany) with 10 K/min and compared to the initial state.

### Thermokinetic analysis and characterisation of the curing progress

DSC measurements at 1, 2, 5, 10, 20 and 40 K/min from 5 to 300 °C provide the basis for the thermokinetic analysis described in Section "Introduction". In this study, the thermokinetic analysis was carried out software-based with *Kinetics Neo* (Erich NETZSCH GmbH & Co. Holding KG, Selb, Germany). The approaches that show the best correlation with the experimental results are chosen from the three thermokinetic methods explained in the introduction. These are applied to predict a temperature profile (max. 5 K/min, max. 180 °C) with a constant conversion rate of 0.25%/min which is in the range of commonly used curing profiles^36,37^. The profiles are linearised for subsequent curing in convection ovens (UF 450 Plus, UF 30, Memmert GmbH + Co. KG, Schwabach, Germany).

Ex-situ *Fourier transform infrared spectroscopy* (FTIR) and DSC measurements are used to validate the predictions. For this purpose, the FTIR Tensor II (Bruker Corporation, Billerica, USA) is used for transmission measurements in the NIR range (7,500 cm^−1^ to 2,500 cm^−1^, resolution 2 cm^−1^, background spectrum: mean value of 40 measurements, sample spectra: mean value of eight measurements). The measurement is performed between two glass slides (Menzel Gläser, Braunschweig, Germany), which are glued together with double-sided adhesive tape (Tesa GmbH, Norderstedt, Germany) forming a measuring cell with a gap of 185 µm. The decrease in the area of the epoxy peak between 4,560–4,497 cm^−1^ at various times of the temperature profile is evaluated. The processing of the raw data includes a baseline correction and a normalisation of the spectra on the constant aromatic peak (4,681 cm^−1^). DSC measurements (25–300 °C, 10 K/min) are performed on several prepared crucibles, which are successively removed from the oven every hour during the run of the material-specific temperature profile. α is determined from the decrease of the exothermic peak.

### Manufacturing, specimen preparation and characterisation of cured resin

After degassing, 4 mm thick plates are manufactured by casting process. Glass transition temperatures (*T*_*g*_*s*) are determined by DSC 204 F1 Phoenix (Erich NETZSCH GmbH & Co. Holding KG, Selb, Germany) according to DIN EN ISO 11,357–2 via midpoint analysis. Therefore, the samples are heated twice from 5C to 300 °C with a heating rate of 10 K/min. Furthermore, *T*_*g*_*s* are determined by *dynamic mechanical thermal analysis* (DMTA) on a GABOEPLEXOR®500N (Erich NETZSCH GmbH & Co. Holding KG, Selb, Germany) in accordance with DIN EN ISO 6721–1 via onset analysis (4 × 8 x 50 mm^3^, 1 Hz, 2 K/min, − 130 °C to 230 °C). *Thermogravimetric analyses* (TGA) are performed according to DIN EN ISO 11,358 at a TGA Q500 (TA Instruments Inc., New Castle, USA) from 20 to 800 °C with 10 K/min for three samples per configuration under nitrogen as well as synthetic air atmosphere.

Mechanical testing was performed according to DIN EN ISO 527–2. For testing matrix material properties, dogbone specimens (1BA) are milled with an EUROMOD®-MP (Isel Germany AG, Eichenzell, Germany), conditioned for 24 h at 40 °C under vacuum and tested on a Z10 universal testing machine from Zwick Roell GmbH und Co. KG (Ulm, Germany) with 5 mm/min. To ensure representativity and reproducibility, all test specimens were extracted from at least two different resin plates at different, defined locations.

## Results and discussion

### Mixture of the components and shelf life of the dispersions

**Figure 1:**
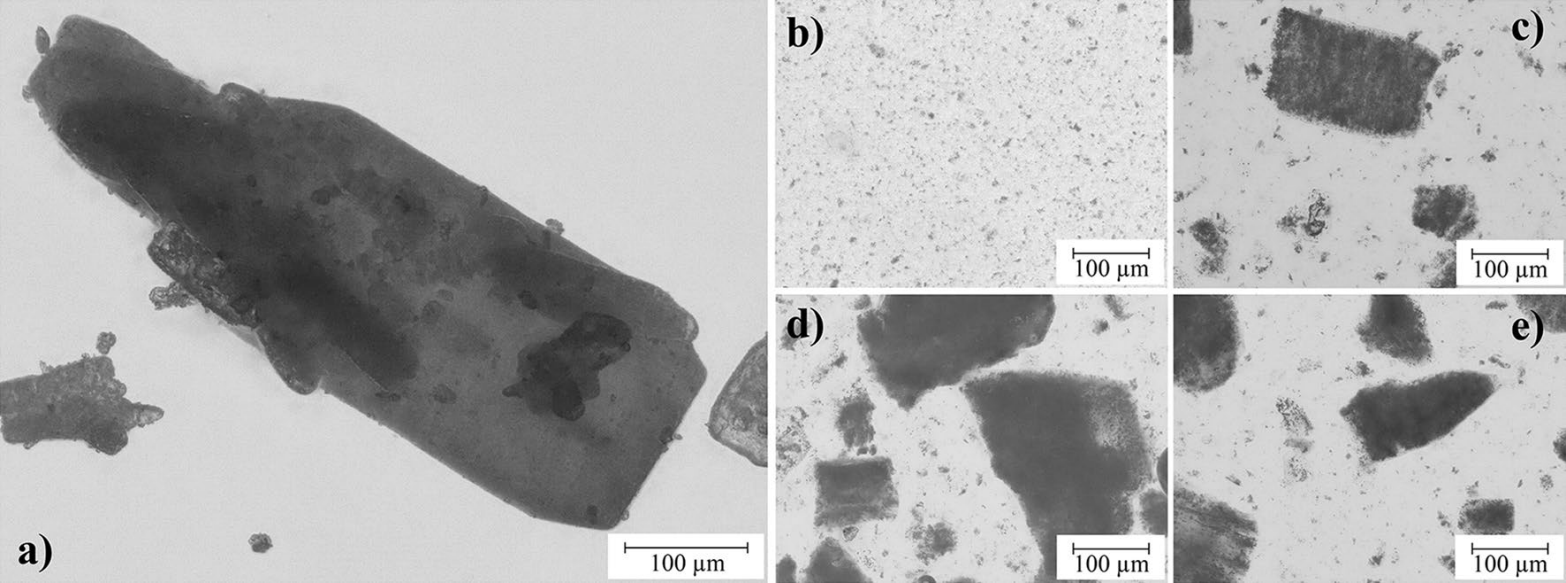
Micoscopy images of L-Arg. (**a**) Initial state as well as dispersed into 827 via (**b**) three-roll mill, (**c**) Thinky-Mixer, (**d**) ultrasonic bath and (**e**) ultrasonic homogeniser.

Exemplary for all configurations, Fig. 1 shows microscopy images of L-Arg (a) and the different dispersion methods in 827 (b–e). Of the methods described in Section "Materials and Mixture of the Components", the three-roll mill (b) is the most suitable for breaking up agglomerates and is therefore used for this study. Due to the minimal gap of 5 µm, the particles are reduced in size and well dispersed. Although the other tested methods (c–e) lead to a reduction in particle size compared to the initial state (a), particles > 100 µm are still present in each case, causing sedimentation and also leaving less particle surface available for interaction with the epoxy resin.

**Figure 2:**
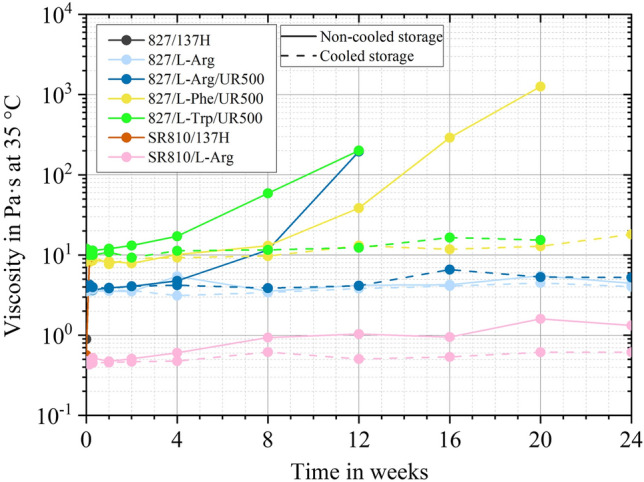
Viscosity in Pa⋅s at 35 °C within 24 weeks for cooled (shaded lines) and non-cooled (dotted lines) storage.

Rheological tests are conducted to determine the flow behaviour of the mixtures of resins and curing agents. In the case of the utilised plate-plate rotational rheometer, torsional shear is applied and the correlation between shear stress and shear rate is determined. The viscosity of thermosets increases as the degree of curing. Repeated measurements over a longer period can be used to determine whether the material shows significant increases in viscosity over time and is therefore not storable under the tested conditions. Figure 2 displays that SR810/L-Arg has a lower viscosity than 827/L-Arg. 827 consists solely of DGEBA, while SR810 also consists partially of DGEBF and 1,4-Butanediol diglycidyl ether, which are less viscous components compared to DGEBA^38^.

Evidently, the viscosities of the resin-hardener dispersions do not increase considerably within 24 weeks of cooled storage. For 827/L-Arg and SR810/L-Arg, it also does not increase significantly in the non-cooled state. In contrast, the curing agent 137H cures both epoxy resins within one day under both storage conditions. As expected, cooling generally extends the shelf-life while the accelerator causes a decrease in latency.

The DSC measurements conducted after 34 weeks confirm the rheology findings. While for 827/L-Arg the enthalpy of reaction in cooled (406.2 J/g) and non-cooled storage (411.7 J/g) do not show any deviations beyond the measuring accuracy compared to the initial state (411.4 J/g), the same curing agent with accelerator shows a different behaviour. The initial reaction enthalpy of 414.1 J/g reduces to 43.8 J/g (equivalent to 89.4% curing) for cooled storage and could not be measured for non-cooled storage because of its high viscosity. Both, the rheological measurements and the DSC results indicate possible applications as prepreg resin systems, whereby energy can be saved by storing prepregs at room temperature or slightly cooled at approx. 11.5 °C compared to the common storage temperature of prepregs (− 18 °C).

### Thermokinetic analysis and characterisation of the curing progress

**Figure 3:**
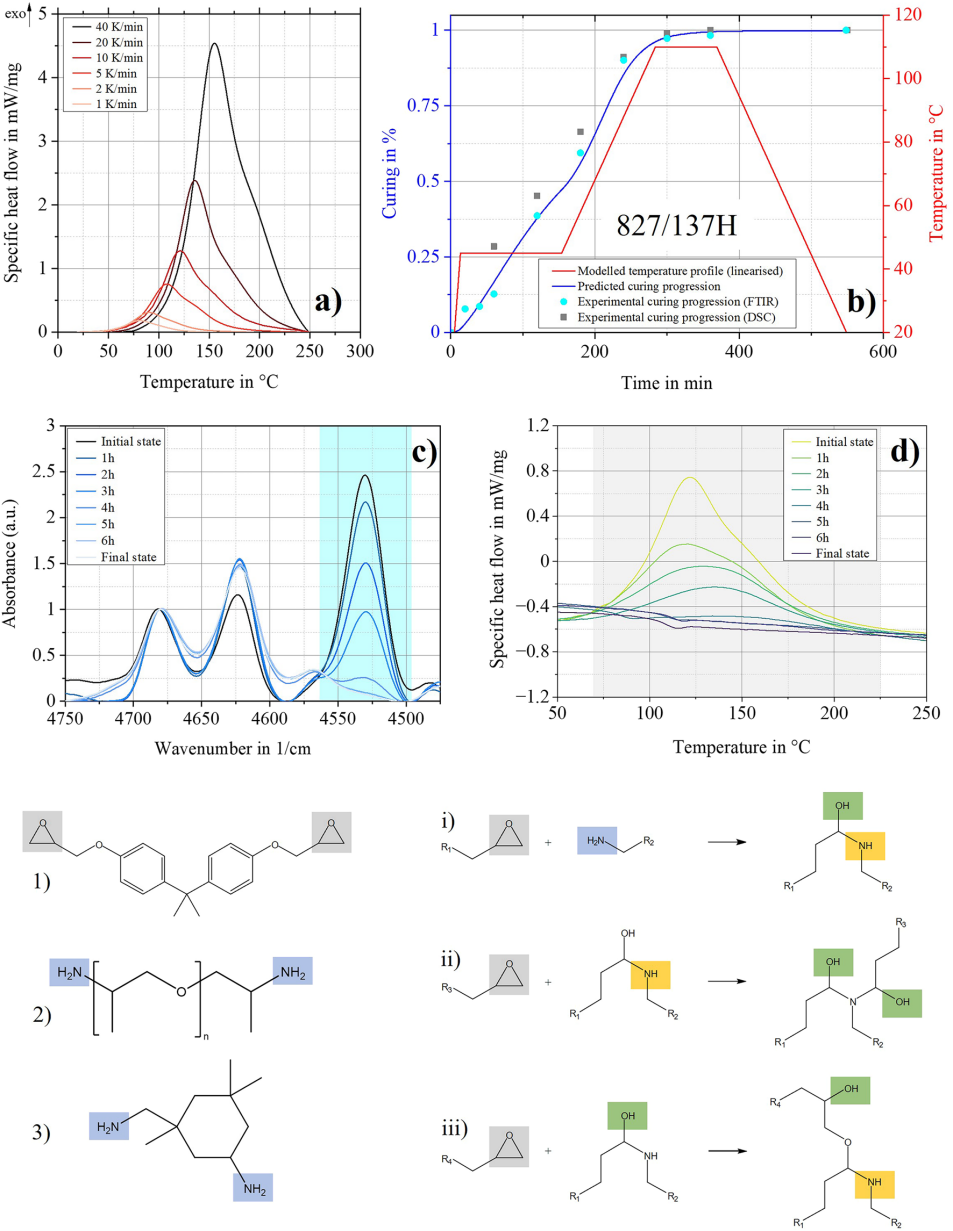
Thermokinetic prediction for the configuration 827/137H. (**a**) DSC thermograms with different heating rates. (**b**) Red shaded line: Linearised, modelled temperature profile, blue shaded line: Related prediction for curing progression and validation with ex-situ measurements: FTIR (blue shaded circles) and DSC (grey shaded squares). (**c**) Decrease of epoxy peak area over time in FTIR spectra during curing—base for blue shaded circles in (b). (**d**) Decrease of reaction enthalpy over time in DSC thermograms during curing—base for grey shaded squares in (b). 1) DGEBA; 2) Poly(oxypropylene)diamine, (3) Isophorondiamine. (i-iii): Occuring reactions, activation energies: (i) 55–60 kJ/mol; (ii) 71 kJ/mol; (iii) 104 kJ/mol^41^.

All curing profiles, thermokinetic methods and the correlation of the experimental data to the respective method can be found as Supplementary Table S2 online. For all configurations, DSC measurements of the cured epoxy resins showed no post-cure peak, which is crucial for verifying complete curing. Figure 3 shows the results for the reference system 827/137H. Figure 3a displays the DSC thermograms of curing at different heat rates as the base for the conducted FM method. Figure 3b shows the linearised temperature profiles achieved with the predicted curing progress (blue, continuous line). With ex-situ FTIR (Fig. 3c) and DSC (Fig. 3d) measurements while curing, the curing progression over time can be shown by analysing the decreasing peak areas. The results are plotted in Fig. 3b for comparison with the prediction. Due to the possibility of normalisation to the aromatic peak, the FTIR measuring points are especially close to the predicted curing curve of the model. The ex-situ DSC measurements are a simple method to monitor the time evolution of the cure with good accuracy and to validate the FTIR results.

The FM method used can map the reactions that take place very well, as these mainly occur sequentially. The two curing agent components Poly(oxypropylene)diamine (Figs. 2, 3) and Isophorondiamine (Fig. 3) contain only primary amino groups, for which the reactions with epoxides (i-iii), including the necessary activation energies, have been well studied. Primary amines react to form secondary amines before these react to form tertiary amino groups. This is visible on the shoulders in the DSC thermograms (Fig. 3a). The esterification (iii) is usually negligible^39,40^.

**Figure 4:**
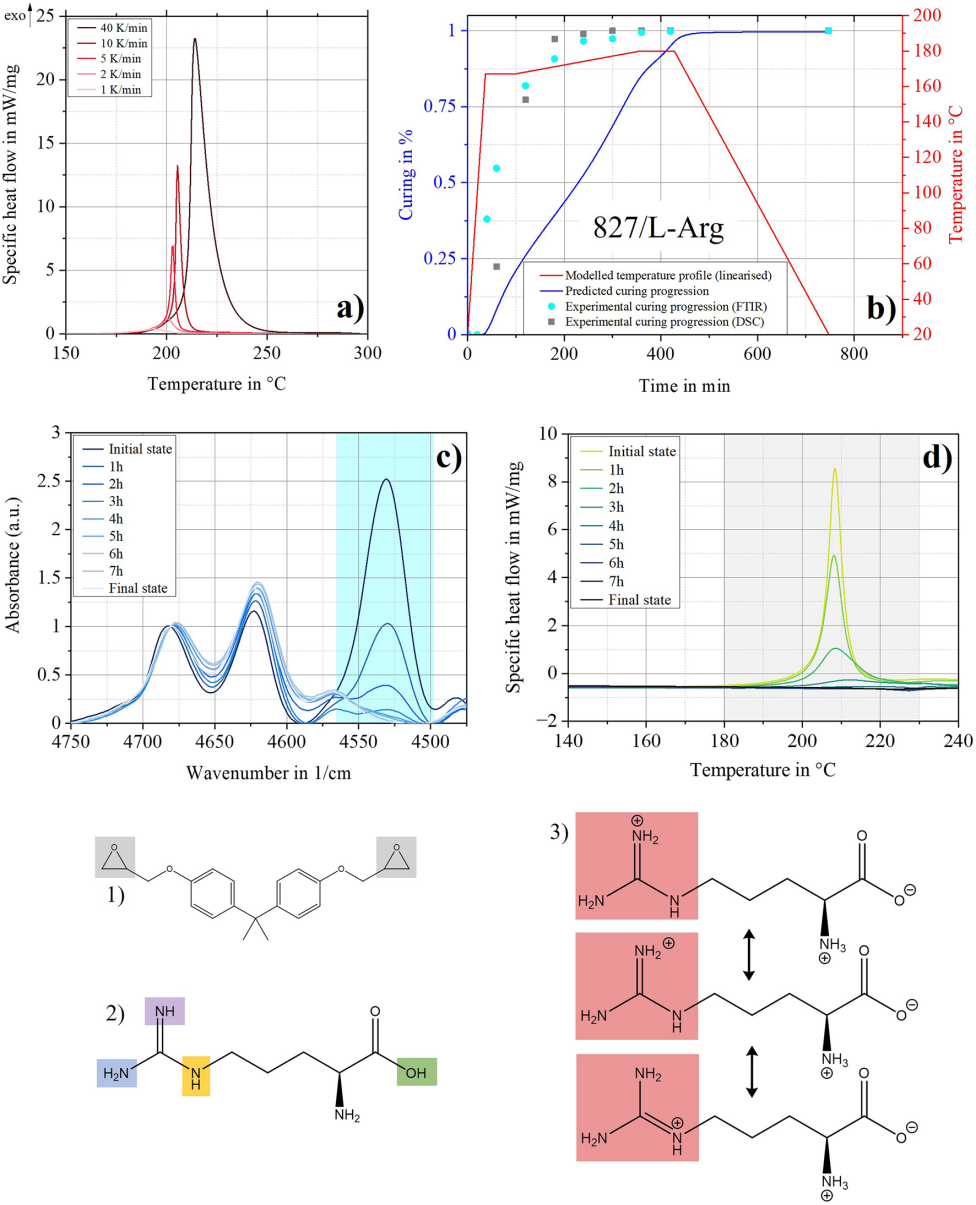
Thermokinetic prediction for the configuration 827/L-Arg. (**a**) DSC thermograms with different heating rates. (**b**) Red shaded line: Linearised, modelled temperature profile, blue shaded line: Related prediction for curing progression and validation with ex-situ measurements: FTIR (blue shaded circles) and  DSC (grey shaded squares). (**c**) Decrease of epoxy peak area over time in FTIR spectra during curing—base for blue shaded circles in (b). (**d**) Decrease of reaction enthalpy over time in DSC thermograms during curing—base for grey shaded squares in (b). (i) and (ii) Areas with varying decreases in epoxy groups and enthalpy consumption. (1) DGEBA; (2) L-Arg; (3) mesomerically stabilised L-Arg.

For 827 cured with L-Arg, the equivalent results are shown in Fig. 4. It can be seen that the used thermokinetic method cannot predict the curing reactions with the same accuracy as for 827/137H. The structural formula of L-Arg (Figs. 2–4) consists of various functional groups that allow diverse reaction mechanisms. The C = N double bond especially offers additional mechanisms. In Fig. 4a, no shoulders can be recognised in the DSC thermograms. Therefore, it can be assumed that the various reactions overlap. If competitive or independent reaction steps occur in addition to consecutive reaction steps, knowledge of the reaction mechanism including the respective reaction order is necessary for adequate predictions. In the considered case, it can also be assumed that the C = N double bond with delocalised π-electrons is mesomerically stabilised as a guanidinium cation (Figs. 3, 4) under the present conditions^42,43^. This further increases the complexity of the occurring reactions.

Historical experiences demonstrate that it takes many years to understand the reaction mechanisms of this complexity reliably. For example, Dicyandiamide (DICY) was commercialised in the 1950s but the first assumption for a precise and complete mechanism was published in 1993^44^. Nevertheless, the findings from the ex-situ DSC and FTIR analysis allow initial assumptions for the reactions of L-Arg with epoxies, on which further investigations can be based. By comparing the exothermic decrease determined by DSC with the decrease of the epoxy groups obtained by the FTIR measurements, it is noticeable that initially ring opening takes place due to fewer exothermic reactions (Fig. 4). Over time, the exothermic reduction logically exceeds the reduction of the epoxy groups, which suggests reactions with higher activation energies, larger exothermic enthalpies or reactions without the presence of the epoxy rings. In addition, it should be mentioned that a perfect fit can be achieved by spline interpolation, but this has no kinetic meaning^45^ and is therefore avoided in this study.

Nevertheless, it can be stated that no more epoxy groups and no more exothermic enthalpy can be detected. Therefore, the determined temperature profile achieves complete curing of the system 827/L-Arg. Curing also follows a constant conversion rate, so further characterisation is carried out with the temperature profiles given in Supplementary Table S2 online. The method used is therefore suitable for creating an initial curing profile for which, the actual conversion processes must be determined with experimental measurements. In the case of 827/L-Arg, the ex-situ measurements demonstrate the potential to carry out the curing at lower temperatures or within shorter times compared to the temperature profile described in Supplementary Table S2 online. This would positively influence composite manufacturing lead times and sustainability due to low energy consumption while manufacturing a product. Suppose the mechanisms are understood in detail in the future, it may be possible to implement more complex models that can reliably represent simultaneous reactions and use targeted accelerators or catalysts to enable curing at lower temperatures.

### Characterisation of cured resin

The glass transition temperatures from DSC and DMTA are summarised in Table 2. The plates were manufactured with the same cooling rate so that an influence of the thermal history on the T_g_ in the first heating run can be excluded. The second heating run shows only small changes within tolerances, which, in addition to the absence of exothermic post-curing peaks in the first heating run, indicates complete curing for all configurations. The curing with L-Arg leads to a T_g_-onset (DMTA) of almost 130 °C and exceeds that of 827/137H by more than 10 K. 827/L-Trp/UR500 also shows a higher T_g_ than the reference system. A dependence of the T_g_ on the resin component can also be shown, SR810 leads to lower *T*_*g*_*s*. As mentioned in Sect. 3.1, SR810 consists partially of DGEBF and 1,4-Butanediol diglycidyl ether as reactive diluent. In addition to lower viscosities^38^ both also generally result in lower *T*_*g*_*s*^46,47^.

**Table 2:** Glass transition temperatures of all configurations obtained from DSC and DMTA.

	*T*_g_ (°C) DSC: Mid-point				*T*_g_ (°C) DMTA: Onset		
827/137H	114.13	±	0.69		118.66	±	0.81
827/L-Arg	121.23	±	0.90		129.05	±	0.38
827/L-Arg/UR500	99.77	±	1.13		106.74	±	0.33
827/L-Phe/UR500	92.87	±	1.19		97.95	±	1.65
827/L-Trp/UR500	113.83	±	1.84		122.86	±	0.46
SR810/137H	90.37	±	0.45		94.56	±	0.77
SR810/L-Arg	84.93	±	3.02		94.55	±	0.33

**Figure 5:**
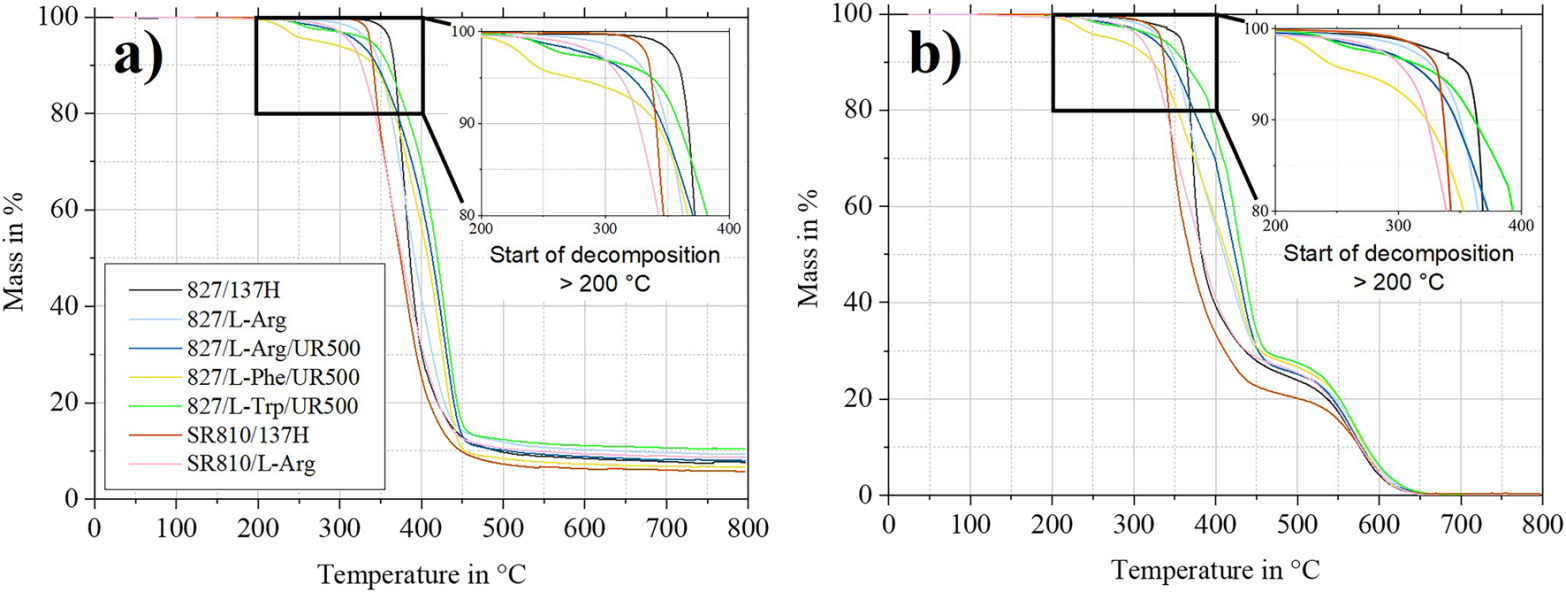
Mass loss curves with detailed view on the onset of mass loss obtained from TGA. (**a**) Under nitrogen atmosphere and (**b**) under synthetic air. One representative graph for each configuration.

TGA can be used to investigate the temperatures at which decomposition processes occur. The decomposition is expressed as a loss of mass. Mass loss curves obtained from the TGA under nitrogen as well as under synthetic air atmosphere are shown in Fig. 5. As expected, residues (8,42 ± 1,82%) are retained under nitrogen atmosphere, while the samples are completely decomposed under synthetic air^48^. Compared to the reference system, the mass loss with the biobased curing agents starts at lower temperatures and extends over a wider temperature range. Therefore, the decomposition temperatures T_z_ (see Supplementary Table S3 online) are at comparable temperatures which are within the normal range of literature values for epoxies^49^. More relevant for practical use is the temperature at which mass loss begins. All configurations demonstrate no mass loss up to 200 °C. By comparing the mass loss curves of the configurations 827/L-Arg and 827/L-Arg/UR500, it is noticeable that the accelerator contributes to a premature onset of the mass loss. After evaluation of the *T*_*g*_*s* and *T*_*z*_*s* it can be stated that the accelerator is not advisable from thermal aspects.

**Figure 6:**
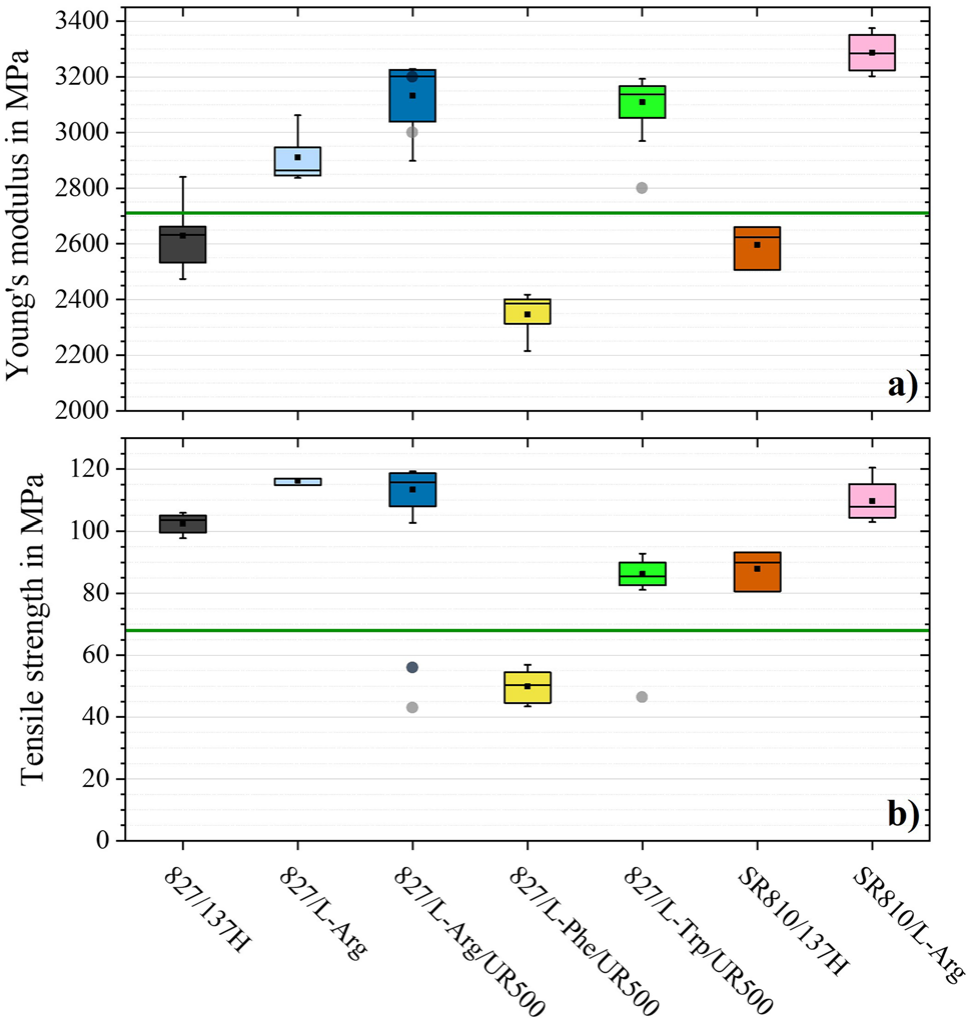
Tensile properties of all configurations. (**a**) Young’s moduli in MPa. (**b**) Tensile strengths in MPa. Literature values for comparison: Grey shaded circles: L-Arg with 1 pph 2-Ethyl-4-methyl-imidazole^6^ and black shaded circles: L-Arg with 1 pph UR500^7^ as accelerator.  Green shaded lines: RIM135/137H as benchmark for conventional epoxy systems^50^.

The results of the tensile tests are displayed in Fig. 6 and summarised in Supplementary Table S4 online. The green lines represent the widely used epoxy resin system RIM135/137H (Westlake, Houston, USA) as an additional reference^50^. Except for the 827-Phe-UR500 configuration, all amino acid-cured epoxy resins show better tensile strengths and moduli. Especially 827/L-Arg achieves excellent tensile strengths and suitable tensile moduli. This can be explained by the fact that L-Arg has the comparatively highest number of reactive sides. This results in a higher cross-linked polymer^6^, which tends to improve tensile strength and modulus^51,52^. In addition, less curing agent is required for a stoichiometric mixing ratio. In contrast to nanoparticles, most microparticles reduce mechanical properties, because larger particles may initiate failure before material defects^53,54^. Therefore, it can be expected that a lower proportion of particles has a less negative effect on the mechanical properties. The influence of the accelerator on the mechanical properties is less than on the thermal properties. With the partially biobased resin SR810, slightly higher tensile moduli and slightly lower tensile strengths are achieved, for which the existing DGEBF content can be mentioned as an explanation^46^.

The values achieved in this study outperform previous literature values for amino acid-cured epoxies^6,7^. The literature values are shown as circles in Fig. 6 and reach significantly worse values in the tensile strengths. The resin component D.E.R. 331 (Olin Epoxy, Stade, Germany) used there is also based on DGEBA and has a very similar EEW, which is why no meaningful influence of the resin component on the achievable properties can be assumed. The reason for the more than twice as high tensile strength with L-Arg is expected to relate to the temperature profiles adapted individually to each configuration in this study. In addition, the ex-situ FTIR and DSC measurements ensured that the temperature profiles resulted in complete curing. Furthermore, it can be particularly highlighted that curing without an accelerator was possible in this study for the same reason.

In addition, the amino acid-cured epoxies produced in this study also offer significantly higher elongations at break (e.g. 827/L-Arg: 5.88 ± 0.29% and 827/L-Arg/UR500: 5.14 ± 0.11% vs. 1.67 ± 0.10%^6^ and 2.65 ± 0.41%^7^), which fulfil the requirements for application in FRP.

## Conclusions

This study presents the development and comprehensive characterisation of biobased cured epoxy as an alternative to state-of-the-art (petrochemical-based) epoxy. In detail, various amino acids (L-Arginine, L-Tryptophan, and L-Phenylalanine) are used as curing agents. The developed epoxies exhibit better thermo-mechanical properties and are considerably more eco-friendly due to non-toxic curing agents. The superiority of the systems presented is based on utilising thermokinetic models to define individual curing profiles.

The highest thermo-mechanical properties are achieved by curing pure DGEBA epoxy resin (827) with L-Arg or L-Trp. With a tensile strength of 116 MPa, an onset glass transition temperature of 129 °C, and a failure strain of almost 6%, L-Arg-cured DGEBA reaches remarkable properties. L-Trp/UR500-cured DGEBA also has an excellent property profile. An onset glass transition temperature of 123 °C, the tensile modulus of more than 3.1 GPa and a tensile strength of 88 MPa are still sufficiently high. In any case, it should be noted that the accelerator UR500 substantially reduces the thermal properties and does not contribute to a significant acceleration of the reactions.

The progress of the reaction was revealed by ex-situ FTIR and DSC measurements. While the predicted curing process for the reference system is in excellent agreement with the model, the prediction deviates from reality for the configuration 827/L-Arg. However, it is demonstrated that the curing occurs at a constant conversion rate and is fully completed in both cases. The complete and complex reaction mechanism has not yet been clarified in the literature. Due to the large number of different functional groups, several different reactions likely co-occur. Therefore, the reactions might exceed the limits of the thermokinetic methods applied. However, the study points out that a deeper understanding of the mechanisms will make it feasible to build up thermokinetic models with better accuracy, select accelerators in a targeted manner and thus reduce reaction temperatures and process durations.

In summary, it was demonstrated that amine acids serve as highly competitive, environmentally, and health-friendly alternatives to petrochemical-based amine curing agents for epoxy resins. The useability of industrial manufacturing processes and the excellent matrix properties represent an advantage over previously developed, bio-based fibre-reinforced plastic composites. Moreover, the study reveals the system's potential for industrial application in prepregs, as they demonstrated a long-term shelf life.

### Supplementary Information

Supplementary Tables.

## Data Availability

Data will be provided by the corresponding author on request.
