# Supplementary material for: Using thermokinetic methods to enhance properties of epoxy resins with amino acids as biobased curing agents by achieving full crosslinking
Source: Sci Rep. 2024 Feb 22;14:4367. doi: 10.1038/s41598-024-54484-0 (PMC10883939; doi:10.1038/s41598-024-54484-0)
Supplement: Supplementary file 1 — Supplementary Tables. [file 41598_2024_54484_MOESM1_ESM.pdf]

Using Thermokinetic Methods to Enhance Properties of Epoxy Resins with Amino Acids as Biobased Curing Agents by Achieving Full Crosslinking

Melissa Walter[1*], Marcel Neubacher[1] and Bodo Fiedler[1]

[1]Hamburg University of Technology, Institute of Polymers and Composites, Hamburg, Germany
[*]melissa.walter@tuhh.de, www.tuhh.de/kvweb

Supplementary Information

Supplementary Table S1: Process parameters and schematic image of the three-roll mill.

Step	Gap A (µm)	Gap B (µm)	n_1 (rpm)	n_2 (rpm)	n_3 (rpm)
1	120	40	33	100	300
2	40	13	33	100	300
3-7	13	5	33	100	300

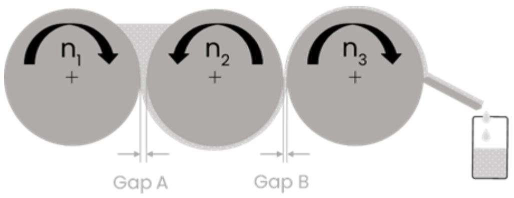

Supplementary Table S2: Linearised temperature profiles and used thermokinetic methods with correlation to the experimental data.

	Temperature profile (°C)	Thermokinetic method	Correlation
827/137H	20 to 45 (3 K/min); 45 (140 min); 45 to 110 (0.5 K/min); 110 (85 min); 110 to 20 (- 0.5 K/min)	FM	0.99
827/L-Arg	20 to 167 (4 K/min); 167 (60 min); 167 to 180 (0.05 K/min); 180 (70 min); 180 to 20 (- 0.5 K/min)	KAS	1.02
827/L-Arg/UR500	20 to 160 (1 K/min); 160 to 180 (0.2 K/min); 180 (90 min); 180 to 20 (- 0.5 K/min)	FM	0.91
827/L-Phe/UR500	20 to 96 (1 K/min); 96 (105 min); 96 to 180 (0.9 K/min); 180 to 20 (- 0.5 K/min)	FM	0.97
827/L-Trp/UR500	20 to 102 (4.8 K/min); 102 (123 min); 102 to 150 (0.5 K/min); 150 (200 min); 150 to 20 (- 0.5 K/min)	FM	0.98
SR810/137H	20 to 37 (0.9 K/min); 37 to 50 (0.1 K/min); 50 to 68 (0.2 K/min); 68 to 95 (0.5 K/min); 95 to 180 (1.9 K/min); 180 (100 min); 180 to 20 (- 0.5 K/min)	FM	1.00
SR810/L-Arg	20 to 160.5 (5 K/min); 160.5 (392 min); 160.5 to 20 (- 0.5 K/min)	FM	0.96

Supplementary Table S3: Decomposition temperatures and respective mass losses in nitrogen and synthetic air atmosphere.

	N_2 atmosphere		Synthetic air atmosphere			
	M_1 (%)	$T_{z,1}$ (°C)	M_1 (%)	$T_{z,1}$ (°C)	M_2 (%)	$T_{z,2}$ (°C)
827/137H	92.25	380.38	76.09	375.53	23.91	569.63
827/L-Arg	90.43	376.38	75.08	367.34	24.92	566.42
827/L-Arg/UR500	91.92	425.12	78.01	419.85	22.00	567.85
827/L-Phe/UR500	88.37	424.47	68.33	424.25	26.43	564.51
827/L-Trp/UR500	89.34	429.89	75.62	436.54	24.38	563.32
SR810/137H	94.55	338.76	78.70	344.00	21.30	569.04
SR810/L-Arg	91.57	362.18	74.05	342.57	25.95	568.18

Supplementary Table S4: Tensile properties of all configurations.

	Tensile strength (MPa)	Young's modulus (GPa)	Elongation at break (%)
827/137H	102.43 ± 2.82	2.63 ± 0.11	10.00 ± 0.74
827/L-Arg	116.22 ± 3.97	2.91 ± 0.08	5.88 ± 0.29
827/L-Arg/UR500	113.30 ± 6.55	3.13 ± 0.14	5.14 ± 0.11
827/L-Phe/UR500	49.93 ± 5.29	2.35 ± 0.07	2.31 ± 0.34
827/L-Trp/UR500	86.22 ± 4.34	3.11 ± 0.08	3.79 ± 0.19
SR810/137H	87.94 ± 5.34	2.60 ± 0.07	9.25 ± 0.85
SR810/L-Arg	109.71 ± 6.67	3.29 ± 0.07	4.94 ± 0.46
